# The Continued Impact of Acute Rejection in the Last Decade of Liver Transplantation

**DOI:** 10.1016/j.gastha.2022.04.021

**Published:** 2022-05-10

**Authors:** C.R. Harrington, T. Bittermann, D. Goldberg, J. Levitsky

**Affiliations:** 1 Division of Gastroenterology and Hepatology, Northwestern University Feinberg School of Medicine, Chicago, Illinois; 2 Division of Gastroenterology, University of Pennsylvania, Philadelphia, Pennsylvania; 3 Department of Epidemiology and Biostatistics, University of Pennsylvania, Philadelphia, Pennsylvania; 4 Division of Gastroenterology, University of Miami, Miami, Florida; 5 Comprehensive Transplant Center, Northwestern University Feinberg School of Medicine, Chicago, Illinois

In 2017, we published a study in *Clinical Gastroenterology and Hepatology* that demonstrated an increased risk of graft failure and death in liver transplant (LT) recipients with acute rejection (AR) in the post-2000 era.^1^ However, an important challenge raised was the recipients’ hepatitis C virus (HCV) status given the frequent diagnostic uncertainty in distinguishing AR from recurrent HCV. This was relevant as that study population originated from an era in which HCV constituted a high percentage of LT recipients with active viremia given the lack of effective direct-acting oral antiviral (DAA) therapies. Nevertheless, the association between AR and inferior recipient outcomes persisted despite stratification by HCV status. Our objective was to re-evaluate the association between AR and post-LT outcomes in the DAA era, in which sustained virological responses are nearly 100%, and thus report data on the impact of AR that is more applicable to current practice.

A retrospective cohort of adult LT recipients was identified using the United Network for Organ Sharing (UNOS) database. Pre-DAA LTs included those performed between 1/1/2010 and 12/31/2013, while the post-DAA period spanned 1/1/2014–12/31/2019. While more recent years of data were available, a substantial increase in missing data with regards to AR was noted beginning 1/1/2020, likely reflecting delays in data reporting and the COVID pandemic. Multiorgan and prior organ transplants were excluded. The last follow-up date for the cohort was 7/1/2021.

AR in the first year post LT was evaluated as a binary exposure. Among patients alive with their native allograft at 1 year post LT, 1-year AR was unable to be determined due to missing data in 18.5% (9183/49,756). All other exposures were measured at LT including sex, age, race/ethnicity, HCV vs non-HCV liver disease, Model for End-stage Liver Disease score, ascites and encephalopathy severity, patient location, dialysis status, history of hepatocellular carcinoma, receipt of living donor LT, receipt of allograft from a donor after circulatory death, and donor age. Primary outcomes of interest included patient and graft survival.

Using a similar approach to the original manuscript,^1^ Cox proportional hazards models evaluated the association of AR with patient and graft survival among patients surviving at least 1 year with their initial allograft, adjusting for the aforementioned covariates. All analyses were performed using STATA v17 (College Station, TX).

Between 1/1/2010 and 12/31/2019, 68,812 LTs were performed, of which 29.1% had HCV. Post-DAA recipients with HCV were older and had less decompensated liver disease and more hepatocellular carcinoma (all *P* < .001; Table A1). HCV recipients had lower rates of 1-year AR than non-HCV recipients in both eras: 11.2% vs 15.5%, respectively, pre-DAA (*P* < .001) and 9.8% vs 13.1% post-DAA (*P* < .001). During the post-DAA era, the etiologies of liver cirrhosis that had the highest rates of 1-year AR era were autoimmune hepatitis (19.3%), primary sclerosing cholangitis (18.3%), primary biliary cholangitis (16.6%), and alcohol-related liver disease (12.8%) (*P* < .001 comparing 1-year AR by diagnosis). Changes in 1-year AR over time were less pronounced in HCV than in non-HCV recipients (*P* = .017 and *P* < .001; Figure A).

**Figure:**
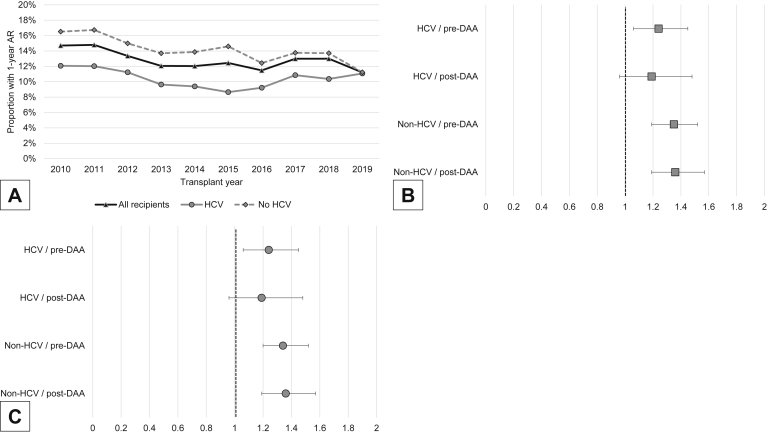
(A) Trends of 1-year AR in HCV vs non-HCV LT over time (2010–2019). (B) HRs of mortality associated with 1-year AR in HCV (pre- and post-DAA), vs non-HCV (pre-and post-DAA). (C) HRs of graft survival associated with 1-year AR in HCV (pre- and post-DAA), vs non-HCV (pre-and post-DAA). AR, acute rejection; DAA, direct-acting oral antiviral; HCV, hepatitis C virus; LT, liver transplant.

Among all patients surviving the first year with their initial liver allograft (89.9%), 1-year AR (adjusted hazard ratio [HR]: 1.30, 95% confidence interval [CI]: 1.21–1.40; *P* < .001) and HCV (adjusted HR: 1.14, 95% CI: 1.08–1.20; *P* < .001) were independent predictors of mortality. This association was also observed with respect to graft survival (adjusted HR: 1.30, 95% CI: 1.21–1.40; *P* < .001) for 1-year AR and (adjusted HR: 1.14, 95% CI: 1.08–1.20; *P* < .001) for HCV. Stratifying by DAA era, HCV was associated with patient survival pre-DAA (adjusted HR: 1.16, 95% CI: 1.08–1.24; *P* < .001), but not post-DAA (adjusted HR: 1.09, 95% CI: 1.00–1.18; *P* = .059). HCV remained a predictor of graft survival pre-DAA and post-DAA, though to a lesser degree (adjusted HR: 1.16, 95% CI: 1.08–1.24; *P* < .001 and adjusted HR: 1.09, 95% CI: 1.00–1.19; *P* = .048, respectively).

In stratified models by HCV status and DAA era, AR during the first year post LT was associated with increased adjusted all-cause mortality in patients with HCV in the pre-DAA era but not in the post-DAA era (adjusted HR: 1.24, 95% CI: 1.06–1.45; *P* = .007 and adjusted HR: 1.19, 95% CI: 0.96–1.48; *P* = .113, respectively). This temporal phenomenon was not observed among non-HCV patients, in whom the risk of mortality associated with 1-year AR remained stable (adjusted HR: 1.35, 95% CI: 1.19–1.52; *P* < .001 and adjusted HR: 1.36, 95% CI: 1.19–1.57; *P* < .001, respectively) (Figure B). Similar findings were obtained for graft survival (Figure C).

In this study, we corroborate our prior study^1^ that AR continues to be a predictor for graft failure and patient death in the non-HCV patient cohort, independent of DAA era. This has also been substantiated in a study by Tanaka et al.^2^ As in our prior study,^1^ this is contrary to data prior to 2000 which suggested that AR had no effect on outcomes.^3^^,^^4^ Ultimately, AR continues to be a significant adverse event, mainly because treatment with high-dose immunosuppression contributes to complications (malignancy, cardiovascular disease, infection, renal dysfunction), likely more so than the impact of AR on the graft itself. These findings may indicate that closer monitoring is required for post-LT patients at higher risk for AR, such as those transplanted for autoimmune liver disease.

Strengths of our study include a large patient population with good generalizability. Limitations of the study include the retrospective nature and missing data in the UNOS database. For instance, we assume that post-DAA patients were cured of HCV, but this cannot be confirmed from this database. Another limitation is that rejection in the UNOS database is based upon provider clinical documentation and entered by transplant coordinators rather than biopsy-proven rejection, making it a less accurate of a measurement. There is also no information on the immunosuppression regimen and compliance of the patients in this dataset. Our power also may be limited since the sample size of HCV recipients was much smaller than non-HCV recipients in the post-DAA era.

In conclusion, as liver failure and need for LT due to HCV is rapidly declining with the introduction of DAA therapy, it is important to focus on the clinically relevant, current population of non-HCV LT recipients. Using updated recent era data, AR continues to be deleterious in the LT population, and avoidance and/or early detection and treatment of AR will likely improve LT outcomes.

## Supplementary Materials

Table A1
