# Supplementary material for: The Continued Impact of Acute Rejection in the Last Decade of Liver Transplantation
Source: Gastro Hep Adv. 2022 May 10;1(5):686–8. doi: 10.1016/j.gastha.2022.04.021 (PMC9681135; doi:10.1016/j.gastha.2022.04.021)
Supplement: Table A1 [file mmc1.docx]

Supplemental Table 1: Clinical characteristics of LT recipients with HCV in the pre-DAA (2010-2013) versus post-DAA (2014-2019) eras

|  | Pre-DAA  2010-2013  (N=8,356) | Post-DAA  2014-2019  (11,642) | p-value |
| --- | --- | --- | --- |
| Male, N (%) | 6,195 (74.1) | 7,788 (75.9) | 0.006 |
| Age (years), median (IQR) | 57 (53-61) | 60 (56-64) | <0.001 |
| Race/ethnicity  White  Black  Hispanic  Asian  Other | 5,777 (69.1)  1,002 (12.0)  1,218 (14.6)  246 (2.9)  113 (1.4) | 7,038 (68.6)  1,188 (11.6)  1,554 (15.1)  301 (2.9)  181 (1.8) | 0.146 |
| Native MELD at LT, median (IQR) | 17 (11-27) | 15 (10-23) | <0.001 |
| Albumin at LT, median (IQR) | 3.0 (2.5-3.5) | 3.2 (2.7-3.8) | <0.001 |
| Ascites at LT, N (%)  None  Mild  Moderate-Severe | 2,189 (26.2)  3,779 (45.2)  2,387 (28.6) | 3,866 (37.7)  4,103 (40.0)  2,291 (22.3) | <0.001 |
| Encephalopathy at LT, N (%)  None  Grade 1-2  Grade 3-4 | 3,314 (39.7)  4,296 (51.4)  745 (8.9) | 4,890 (47.7)  4,572 (44.6)  798 (7.8) | <0.001 |
| HCC, N (%) | 4,017 (48.1) | 6,585 (64.2) | <0.001 |
| LDLT, N (%) | 239 (2.9) | 297 (2.9) | 0.890 |
| DCD liver, N (%) | 466 (5.6) | 739 (7.2) | <0.001 |
| Donor age (years), median (IQR) | 41 (27-52) | 41 (29-54) | <0.001 |
