# Supplementary material for: The Continued Impact of Acute Rejection in the Last Decade of Liver Transplantation
Source: Gastro Hep Adv. Author manuscript; Available in PMC 2022 Nov 22. (PMC9681135; doi:10.1016/j.gastha.2022.04.021)
Supplement: Supplementary file 1 [file NIHMS1835131-supplement-1.docx]

Supplemental Table A1: Clinical characteristics of LT recipients with HCV in the pre-DAA (2010-2013) versus post-DAA (2014-2019) eras

|  | Pre-DAA 2010-2013 (N=8,356) | Post-DAA 2014-2019 (11,642) | p-value |
| --- | --- | --- | --- |
| Male, N (%) | 6,195 (74.1) | 7,788 (75.9) | 0.006 |
| Age (years), median (IQR) | 57 (53-61) | 60 (56-64) | <0.001 |
| Race/ethnicity      White      Black      Hispanic      Asian      Other | 5,777 (69.1) 1,002 (12.0) 1,218 (14.6) 246 (2.9) 113 (1.4) | 7,038 (68.6) 1,188 (11.6) 1,554 (15.1) 301 (2.9) 181 (1.8) | 0.146 |
| Native MELD at LT, median (IQR) | 17 (11-27) | 15 (10-23) | <0.001 |
| Albumin at LT, median (IQR) | 3.0 (2.5-3.5) | 3.2 (2.7-3.8) | <0.001 |
| Ascites at LT, N (%)      None      Mild      Moderate-Severe | 2,189 (26.2) 3,779 (45.2) 2,387 (28.6) | 3,866 (37.7) 4,103 (40.0) 2,291 (22.3) | <0.001 |
| Encephalopathy at LT, N (%)      None      Grade 1-2      Grade 3-4 | 3,314 (39.7) 4,296 (51.4) 745 (8.9) | 4,890 (47.7) 4,572 (44.6) 798 (7.8) | <0.001 |
| HCC, N (%) | 4,017 (48.1) | 6,585 (64.2) | <0.001 |
| LDLT, N (%) | 239 (2.9) | 297 (2.9) | 0.890 |
| DCD liver, N (%) | 466 (5.6) | 739 (7.2) | <0.001 |
| Donor age (years), median (IQR) | 41 (27-52) | 41 (29-54) | <0.001 |
